# Abstract of Cases Reported at Meeting of Tri-State Medical Association, October 12-15, 1896

**Published:** 1896-11

**Authors:** R. R. Kime

**Affiliations:** Atlanta, Ga.


### ABSTRACT OF CASES REPORTED AT MEETING OF TRI-STATE MEDICAL ASSOCIATION, OCTOBER 12 TO 15, 1896.

By R. R. KIME, M.D.,

ATLANTA, GA.

CASE 1.—A unicervical septus uterus with abortion from right side; fetal structures had passed off, the decidual structures retained, infected; removed them, disinfected and drained. Right side measured three and one-half inches in depth, left side, nongravid, measured one a half inches in depth.

CASE 2.—Uterine hemorrhage, eight days after delivery, due to typhoid fever commencing at labor. Uterus had to be tamponed for a period of three weeks, changing every one to two or three days, as demanded by symptoms. The tampon each time had to be quickly replaced to prevent patient bleeding to death.

CASE 3.—One of placenta previa centralis, with infection before delivery, which had been having hemorrhage four or five days when first seen. Immediately delivered by podalic version; peeled off placenta, irrigated and disinfected uterus, dressing patient antiseptically. Within two days symptoms of infection returned with renewed vigor, with severe pain and elevated temperature, which the physician in charge tried to control by morphia and phenacetin, to no avail. Visiting patient again, the author immediately administered salines, irrigated, disinfected and drained uterus, and in a few hours patient was comparatively easy; no more opiates or coal-tar derivatives given. The drainage (tubular) kept up six days, at which time patient was practically convalescent.

CASE 4.—Tumor and infection complicating labor. Infection treated by irrigation and tubular drainage, tumor disappearing by absorption. Patient examined a year afterward and no evidence of tumor, which was probably a fibroid, and situated in posterior wall of uterus.

CASE 5.—Was one of extreme septic intoxication, seen ten days after labor, in which the utility of tubular drainage was demonstrated. When tube was removed, constitutional symptoms returned, which could only be controlled by reinserting tube. Gauze failed to meet the indications.

